# Emergency department mental health presentations before and during the COVID-19 outbreak in Western Australia

**DOI:** 10.1177/1039856220960673

**Published:** 2020-09-22

**Authors:** Milan Dragovic, Viki Pascu, Tammy Hall, Jesse Ingram, Flavie Waters

**Affiliations:** North Metropolitan Health Service, Mental Health, Clinical Research Centre, Australia; Division of Psychiatry, School of Medicine, the University of Western Australia, Australia; North Metropolitan Health Service, Mental Health, Clinical Research Centre, Australia; North Metropolitan Health Service, Mental Health, Clinical Research Centre, Australia; North Metropolitan Health Service, Mental Health, Clinical Research Centre, Australia; North Metropolitan Health Service, Mental Health, Clinical Research Centre, Australia; School of Psychology, the University of Western Australia, Australia

**Keywords:** emergency department, mental health, COVID-19, coronavirus

## Abstract

**Objective:**

Coronavirus (COVID-19) has led to high levels of psychological distress in the community. This study aimed to examine whether emergency departments (EDs) also recorded a rise in mental health presentations.

**Method:**

Changes in the number, and type, of mental health presentations to Western Australia EDs were examined between January and May 2020, and compared to 2019.

**Results:**

Data showed an unexpected decrease in the number of mental health presentations, compared to 2019, which was temporally coincident with the rise in local COVID-19 cases. Presentations for anxiety and panic symptoms, and social and behavioural issues, increased by 11.1% and 6.5%, respectively, but suicidal and self-harm behaviours decreased by 26%.

**Conclusion:**

A rise in local COVID-19 cases was associated with a decrease in mental health presentations to EDs. This has important implications for the planning and provision of healthcare services in the current pandemic.

Recent reports about the mental health impact of the COVID-19 pandemic have revealed a need for services to understand how the population is utilising and accessing healthcare and emergency services.^1^ Increased levels of psychological distress, including post-traumatic stress, major depression and suicide, are commonly reported after mass disasters and pandemics.^2,3^ Similarly, a recent meta-analysis of studies conducted during the COVID-19 pandemic has revealed high levels of stress, anxiety and depression in the community,^4^ possibly exacerbated by extensive media coverage and the daily reporting of new cases and deaths.^5^

These observations have led to speculations about a possible surge in the prevalence of anxiety, depression, suicide, and harmful use of drugs and alcohol,^6,7^ and the need to prepare for possible congestions of healthcare systems, and especially emergency departments (EDs). In responses for calls from international health organisations, the Australia Government released funding to improve the capacity of emergency and healthcare services to prepare for an imminent mental health crisis.

A lack of data exists, however, regarding healthcare service uptake during COVID-19. Health behaviours are complex, and service access is influenced by multiple factors including an individual’s risk assessment and other social factors.^8^ During the pandemic, individuals were required to balance their need for urgent mental healthcare against the risk of infection in hospital settings, possibly impacting on help-seeking behaviour.

This study examined the number and characteristics of ED presentations for mental health reasons to Western Australia (WA) Metropolitan hospitals. WA recorded 620 cases in the first 6 months of 2020 and nine deaths, with early restrictions helping to combat the outbreak with few recurrences. We examined week-by-week change in hospital ED presentations between the start of the COVID-19 outbreak and until the easing of restrictions, and compared the results against the same period in 2019.

## Methods

Mental health attendances to three WA North Metropolitan Health Services EDs were extracted from the Emergency Department Data Collection database. The area population comprises approximately 800,000 residents,^9^ with mental and behavioural disorders representing 5% of all ED presentations.^10^ Collection dates ranged from 25 January to 1 May for 2 consecutive years (2019 and 2020), corresponding to the first case of COVID-19 in Australia until the easing of restrictions. Incomplete weeks (beginning and end of periods) were excluded from analysis. Variables extracted included patient demographics, ED service usage (admissions, transfers, discharges and length of stay), reasons for presentation (coded symptom descriptors) and most common principal psychiatric diagnoses (ICD-10, comprising 92.4% of cases with a diagnoses), using standardised coding.^11^ Frequent attendees were defined as individuals presenting four or more times within the study period.

Data were analysed using the statistical package Jamovi. The number and percentages of attendances and individual persons are reported, and groups were compared with independent samples *t*-test or χ^2^ test. Approvals were provided by the North Metropolitan Health Service (NMHS) Quality Assurance and Evaluation with the intent to publish with Human Research Ethics Committee overview.

## Results

Data included 7140 attendances (5522 persons) over the two study periods (Table 1). Compared to 2019, ED data from 2020 showed significantly fewer presentations, and individual cases (including frequent attendees) (*p* < .001). There was no difference in age or gender, but Aboriginal and Torres Strait Islander people were less likely to present to EDs in 2020 (from 4.4% to 2.8%). In addition, the 2020 data showed significantly reduced length of stay (5.6 h vs 6.7 h, *p* < .001), and a 2.4% reduction in the use of private transport (*p* = .042) with an equivalent increase in ambulance use (*p* = .041). There were also (non-significant) reductions in non- and semi-urgent presentations (1.4%), with a corresponding increase in more urgent triage categories, and individuals were less likely to wait to be attended.

**Table 1.** Demographic and hospital attendance profile of patients presenting to an ED for a mental health reason in January–May 2019 and 2020

Demographic variables	2019	2020	Test values	*p* value
Presentations	3,674	3,466	215.5	<.001
People	2,826	2,696	133.0	<.001
Age, ***M*** (***SD***)	39.1 (20.2)	39.5 (20.6)	.67	.504
Sex, male, ***n*** (%)	1688 (45.9)	1573 (45.4)	.18	.672
Aboriginal or Torres Strait Islanders, ***n*** (%)				
**Attendances**	216 (5.9)	162 (4.7)	5.10	.024
**People**	164 (6.3)	98 (4.7)	5.61	.018
Triage classification (***n***)				
1 - **Resuscitation**	39 (1.1)	51 (1.5)	2.23	.135
** 2 - Emergency**	411 (11.2)	405 (11.7)	.44	.507
** 3 - Urgent**	1832 (49.9)	1746 (50.4)	.18	.672
** 4 - Semi-urgent**	1282 (34.9)	1144 (33.0)	2.87	.090
**5 - Non-urgent**	110 (3.0)	120 (3.5)	1.42	.233
Length of stay (h), *M (SD)*	6.7 (13.0)	5.6 (5.9)	4.56	<.001
NEAT target, ***n*** (%)	1858 (48.4)	1980 (51.6)	7.30	.01
Mode of arrival				
**Private transport**	1774 (48.3)	1590 (45.9)	4.12	.042
**Ambulance**	1601 (43.6)	1594 (46.0)	4.15	.041
**Police**	256 (7.0)	231 (6.7)	.25	.616
**Other**	43 (1.1)	51 (1.4)	1.30	.253
Referral self/relative, ***n*** (%)	2994 (81.5)	2818 (81.3)	.04	.837
Departure status				
**Departed under own care**	1860 (50.6)	1816 (52.4)	2.31	.128
**Admitted to ED**’**s Obs ward**	967 (26.3)	874 (25.2)	1.13	.288
**Admitted to ward**	344 (9.4)	373 (10.8)	3.86	.049
** Transferred to another**	309 (8.4)	282 (8.1)	.21	.645
**Hospital**				
** Did not wait to be attended**	126 (3.4)	75 (2.2)	9.38	.002
** Left at own risk**	51 (1.4)	39 (1.1)	1.30	.255
** Nursing home**	17 (0.5)	7 (0.2)	4.54	.033

*Note.* ED = emergency department, M = mean, NEAT = National Emergency Access Target, Obs = observation, SD = standard deviation.

**Figure 1.**
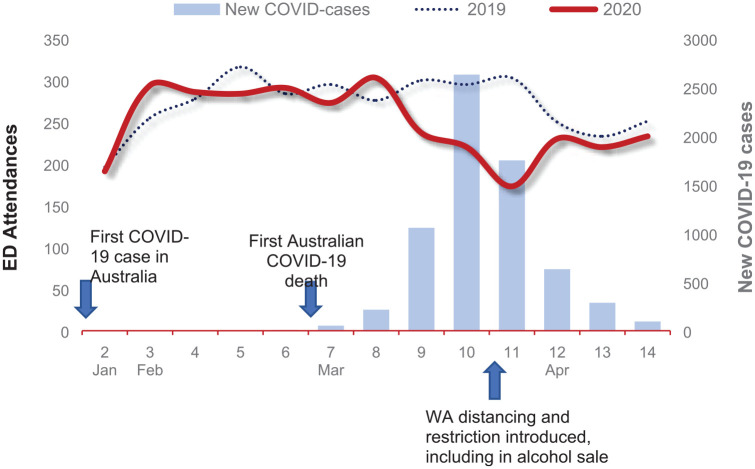
Weekly percentage variance of ED presentations for a mental health reason in the first 14 weeks of 2019 and 2020, including weeks 7–14, which recorded the first death in WA and Australia and the gradual increase in social distancing and other restrictions. ED = emergency department.

Figure 1 shows a rapid decline in ED attendances, which is aligned with the rise in COVID-19 cases. The change in presentation closely follows the first COVID-19 death in one of these hospitals, and the beginning of restrictions (on 9 March). In Week 10, there was a 43% reduction in ED presentations compared to 2019.

In 2020, the most common psychiatric diagnoses were neurotic and stress-related disorders (41.9%), substance-related disorders (22.2%), followed by schizophrenia-spectrum disorders (11.0%), affective disorders (9.2%) and other organic mental disorders (8.1%). Table 2 lists the most common mental health complaints and reasons for ED presentation. Compared to 2019, presentations due to anxiety/panic symptoms and social/behavioural issues increased by approximately 10%, but there were significant reductions across all other complaint types. Suicidality and self-harm behaviours, usually the most common reasons for ED presentations in previous years, dropped by 26%. In addition, the proportion of drug-related presentations increased from 47% to 56%, while alcohol-related presentation declined from 53% to 44%.

**Table 2.** Most common (mental health) presenting complaints in ED at the height of COVID-19 epidemic and compared to the equivalent period in 2019

Top 15 presenting complaints (91.3% of all complaints during the 4 weeks in 2020)	2019 (weeks 8–11) *n*	2020 (weeks 8–11) *n* (% variation)	χ^2^ test	*p* value
Suicidal\ behaviour	269	199 (↓ 26.0%)	80.2	<.001
Social/behavioural problems	77	82 (↑ 6.5%)	5.5	.02
Anxiety/panic symptoms	54	60 (↑ 11.1%)	6.3	.01
Inappropriate behaviour	59	43 (↓ 27.1%)	17.9	<.001
Depressed	55	42 (↓ 23.6%)	14.3	<.001
Deliberate self-harm	73	40 (↓ 45.2%)	42.3	<.001
Alcohol intoxication	48	32 (↓ 33.3%)	19.0	<.001
Drug overdose	24	24 (0.0%)	-	-
Hallucinations	28	21 (↓ 25.0%)	7.9	.01
Psychiatric problems	36	21 (↓ 41.7%)	18.7	<.001
Violent/aggressive behaviour	24	20 (↓ 16.7%)	4.3	.04
Drug abuse	31	19 (↓ 38.7%)	25.3	<.001
Requesting psychiatric review	38	18 (↓ 52.6%)	26.8	<.001
Situational crisis	25	17 (↓ 32.0%)	9.3	<.01
Social problems	14	16 (↑ 14.3%)	2.1	.14

*Note.* ED = emergency department.

## Discussion

The current findings showed significant reductions in mental health-related ED presentations, compared to 2019. This does not support early speculations about a possible surge in mental health service access, although it is aligned with early observations in Europe.^12–14^ In this report, a unique finding is the demonstration of a temporal coincidence in the decline of ED presentations, which coincided with the growth of new COVID-19 cases and which points to a causal epidemiological association.^15^

Our analysis of patient characteristics and service access suggested that a number of social and individual factors influenced service access.^8^ Reductions in the number of vulnerable groups such as indigenous people as well as reductions in waiting time and in the use of private transport suggest a fear of contagion from hospitals settings.^16–18^ These may be viewed as high-risk locations for contracting the disease,^16^ especially given media coverage about hospital systems being overwhelmed.

Another factor possibly involved government guidance. Quarantine, travel restrictions and social distancing likely contributed to significant reduction in presentations and the use of private transport. Reduction of alcohol-related presentations also coincides with WA government’s limits in the sale of alcohol to curb alcohol-related violence and damage to health.

The current finding of reduced ED presentations is unlikely to reflect a lowered prevalence of mental illness in the community given the results of recent population surveys, and therefore raise questions about coping mechanisms of individuals in crisis. It is likely that alternative services were accessed. For example, calls to mental health helplines increased by 30%,^19,20^ suggesting that individuals found other ways to access care without travelling to EDs. Given evidence suggesting that helpline and telephone counselling services have a positive impact on suicide ideation and prevention,^21–23^ the recent release of government funding to community organisations is much needed. Nonetheless, service options for people in crisis must be reviewed taking into account the individual and social restrictions imposed by the pandemic.

Current limitations included a focus on ED, which is not representative of other mental health support services and all population groups. Second, the results are difficult to extrapolate to other states in Australia, given the relatively privileged position of WA due to the early implementation of strict restriction measures and small population density. Finally, the results point to a temporal association between individual and societal changes due to COVID-19 and reductions in ED presentations, but causal extrapolation cannot be conclusively determined. While t*emporality* is an essential criterion in determining causality,^15^ other criteria such as strength of association^24^ require independent examination.

In conclusion, indicators of community psychological distress in the early months of COVID-19 did not correspond to an increase in ED mental health presentations in WA. Future research is required to better understand whether this surge has simply been delayed or rechannelled via other routes. Regarding the surge of anxiety amongst young people and those who are experiencing strong lockdown measures, additional mental health services and/or subsidised psychological counselling should be provided.
